# Exogenous overexpression of nerve growth factor in the urinary bladder produces bladder overactivity and altered micturition circuitry in the lumbosacral spinal cord

**DOI:** 10.1186/1472-6793-7-9

**Published:** 2007-08-28

**Authors:** Peter Zvara, Margaret A Vizzard

**Affiliations:** 1 Department of Neurology, University of Vermont College of Medicine, 89 Beaumont Avenue, Burlington, VT 05405, USA; 2 Department of Anatomy and Neurobiology, University of Vermont College of Medicine, 89 Beaumont Avenue, Burlington, VT 05405, USA; 3 Department of Surgery, University of Vermont College of Medicine, 89 Beaumont Avenue, Burlington, VT 05405, USA

## Abstract

**Background:**

Exogenous NGF or saline was delivered to the detrusor smooth muscle of female rats for a two-week period using osmotic mini-pumps. We then determined: (1) bladder function using conscious cystometry; (2) organization of micturition reflexes using Fos protein expression in lumbosacral (L5-S1) spinal cord neurons; (3) calcitonin gene-related peptide (CGRP)-immunoreactivity (IR) in lumbosacral spinal cord segments.

**Methods:**

An osmotic pump infused 0.9% NaCl (n = 6) or NGF (n = 6)(2.5 μg/μl solution; 0.5 μl/hr) for two weeks into the bladder wall. NGF bladder content was determined by enzyme-linked immunoassays. Bladder function was assessed with conscious cystometry. Immunohistochemical and imaging techniques were used to determine the distribution of Fos-IR cells and CGRP expression in the L5-S1 spinal cord in saline and NGF-treated rats two hours after intravesical saline distention. Fos expression and CGRP-IR in NGF-treated rats with bladder distention was compared to that observed in cyclophosphamide (CYP; 75 mg/kg; i.p.) treated rats with bladder distention.

**Results:**

Two-week infusion of NGF into the bladder wall increased bladder weight, reduced bladder capacity (60%), reduced the intercontraction interval (60%) and increased the amplitude of non-voiding contractions. NGF treatment and intravesical saline distention (2 hr) increased expression of Fos protein in L6-S1 spinal cord and altered the distribution pattern of Fos-IR cells. CGRP-IR in the lumbosacral spinal cord was also increased after NGF treatment.

**Conclusion:**

These data suggest that NGF infusion into the bladder wall induces bladder overactivity, can reveal a "nociceptive" Fos expression pattern in the spinal cord in response to a non-noxious bladder stimulus and increases CGRP-IR in the lumbosacral spinal cord.

## Background

The exact contribution of nerve growth factor (NGF) to bladder function is not known but a correlation between bladder overactivity and elevated urinary bladder NGF has been suggested. Experimentally-induced cystitis induces bladder overactivity and increases bladder NGF protein and transcript. In the spontaneously hypertensive rat model, increased expression of bladder NGF is associated with hyperactive voiding [1]. Bladder outlet obstruction is associated with increased expression of bladder NGF [2-4]. Intrathecal [5], intravesical delivery of NGF [6] or adenovirus-mediated NGF overexpression [7] in the bladder induces bladder overactivity and bladder afferent cell hyperexcitability [5,8] in control rats. NGF scavenging methods [6,9] reduce bladder overactivity in rats with experimentally induced inflammation. Elevated levels of neurotrophins are detected in the urine of women with painful bladder syndrome [10] (PBS)/interstitial cystitis (IC) or in the urothelium of individuals with neuropathic bladder [11]. However, a recent study failed to demonstrate an association between increased urothelium/suburothelium NGF with detrusor overactivity or bladder sensation [12].

Neurotrophins, through interactions with Trk and/or p75^NTR ^receptors, may contribute to neurochemical [13,14], electrophysiological [8] and organizational [4,15] plasticity of lower urinary tract (LUT) pathways after cystitis. Neurotrophin/Trk and/or p75^NTR ^interactions could induce long-term changes in cells [4], including: (1) mediating neurotransmitter phenotype; (2) influencing dendrite size and synaptic reorganization; (3) increasing synaptic efficacy; and (4) controlling innervation density and target organ function.

In the present study, exogenous NGF or saline was delivered to the detrusor smooth muscle for a two-week period using osmotic mini-pumps. We then determined: (1) bladder function using conscious cystometry; (2) organization of micturition reflexes using Fos protein expression in lumbosacral (L5-S1) spinal cord neurons and (3) calcitonin-gene related peptide (CGRP) immunoreactivity (IR) in the lumbosacral spinal cord.

## Methods

Female Wistar rats (Charles River Canada) were housed individually on a 12 hr light/12 hr dark cycle. Protocols were approved by UVM IACUC (06–014), under the supervision of Office of Animal Care in accordance with AAALAC and NIH guidelines.

### Animal surgery

**Figure 1:**
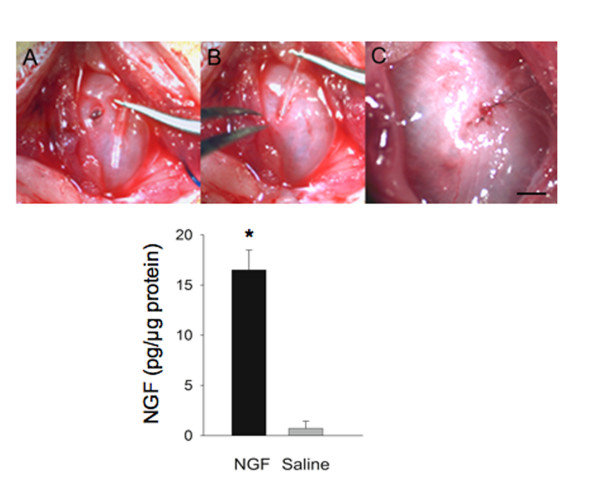
**Intraoperative procedure for tubing implantation**. Intraoperative picture depictingthe insertion of PE10 tubing into the urinary bladder wall. Bundles of detrusor muscle are dissected from the urothelium (**A**). A 4–5 mm length of tubing is inserted into the bladder wall (**B**). Tubing is secured in place by two 10-0 nylon sutures (**C**). Significant (p ≤ 0.001) increase in total urinary bladder nerve growth factor (NGF; **D**) as detected with ELISA after two-week exogenous delivery of NGF to bladder wall. *, p ≤ 0.001. 'n' = 6 for each group in **D**.

An osmotic pump (Alza Corporation) infused 0.9% NaCl (n = 6) or NGF (n = 6)(2.5 μg/μl solution; 0.5 μl/hr)(Genentech, Inc.) for two weeks. This NGF concentration and release rate were previously used in chronic, intrathecal administration of NGF and resulted in bladder overactivity and hyperexcitability of bladder afferents [5]. Under general anesthesia, the urinary bladder was exposed through a lower midline abdominal incision. The osmotic pump was secured subcutaneously in left upper quadrant of the abdomen. Biocompatible silicon adhesive (Kwik Sil, World Precision Instruments) was used to attach the proximal end of PE10 tubing (Clay Adams Parsippany, NJ) to the osmotic pump. The distal end of the tubing was brought into the abdominal cavity and tunneled between the detrusor and bladder adventitia. Two 10-0 nylon sutures secured the tubing in the bladder wall. (Fig. 1A–C)

Twelve days after pump implantation, PE50 tubing with the end flared was inserted into the dome of the bladder and secured with a 6-0 nylon purse string suture. The distal end of the tubing was sealed, tunneled subcutaneously and externalized [9].

### CYP-treatment

A group of rats (n = 4) treated with CYP (75 mg/kg every 3^rd ^day; i.p.) for ten days had intravesical catheters implanted as described above on day 8.

### Cystometrograms

Forty-eight hours after tube implant, rats were placed unrestrained in a metabolic cage [9]. The bladder catheter was connected via a T-tube to a pressure transducer and microinjection pump. Room temperature saline solution (0.9%) was infused (10 ml/hr) into the bladder and the animal urinated into a pan on top of a balance [9]. At least three reproducible micturition cycles were recorded (LabVIEW, National Instruments) and the following recorded [9]: filling pressure (FP – pressure at the beginning of bladder filling), threshold pressure (TP – bladder pressure immediately prior to micturition), micturition pressure (MP – the maximal bladder pressure during micturition), presence or absence of non-voiding bladder contractions (NVCs – increases in bladder pressure at least 10 cm H_2_O without release of urine). The post-void residual (PVR) was measured by aspirating the urine remaining in the bladder after the last micturition or draining the bladder by gravity. Bladder capacity (BC) was calculated as the sum of voided volume and PVR. Compliance was defined as the mean rise in intravesical pressure (cm H_2_O/ml) measured at 0–20% of capacity.

### Euthanasia and tissue handling

Two hours after infusion [15], animals were anesthetized and killed by intracardiac perfusion with oxygenated Krebs buffer (pH 7.4) (95% O2, 5% CO2) followed by 4% paraformaldehyde. After perfusion, the spinal cords were dissected, postfixed, rinsed in phosphate buffered saline (PBS; 0.1 M NaCl in phosphate buffer, pH 7.4) and placed in sucrose solutions (10–30%) in 0.1 M PBS for cryoprotection. Spinal cord segments (L5-S1) were sectioned (40 μm) on a freezing microtome. Spinal cord segments were identified based upon at least two criteria: (1) the T13 dorsal root ganglion exits after the last rib and (2) the L6 vertebra is the last moveable vertebrae followed by the fused sacral vertebrae.

### Preparation of ELISA samples

Prior to perfusion, the urinary bladder was dissected and weighed. Individual bladders were solubilized in Tissue Protein Extraction Reagent (Pierce Biotechnology, Woburn, MA) with protease inhibitors (Roche Diagnostics GmbH, Germany). Tissue was homogenized and centrifuged (10,000 rpm for 5 min); supernatants were used for NGF quantification [16]. Total protein was determined (Pierce). The NGF E-max immunoassay system (Promega Corp., Madison, WI) demonstrates very low cross-reactivity with structurally related growth factors. The NGF standard generated a linear standard curve from 7.8–500 pg/ml (r^2 ^= 0.997, p ≤ 0.001). Absorbance values of standards and samples were corrected by subtraction of background value. Samples were diluted to bring the absorbance values onto the linear portion of the standard curve. Curve fitting of standards and evaluation of NGF content of samples was performed using a least squares fit.

### c-Fos immunohistochemistry

Spinal cord sections (L5-S1) were incubated for 72 hours at 4°C with c-Fos antisera (1:5K; Calbiochem, San Diego, CA) diluted in potassium PBS (KPBS) plus 0.4% Triton X-100. The antibody was visualized with an avidin-biotin horseradish peroxidase complex (Vector Laboratories, Burlingame, CA). Tissue sections were mounted on slides, dehydrated, cleared in xylene, coverslipped with Permount and examined with brightfield microscopy [15]. Tissue from sham or NGF groups was processed simultaneously. Control tests run without antisera or with antisera preabsorbed with Fos protein eliminated Fos staining.

### Quantification and statistical analysis

The number of cells exhibiting Fos-IR was estimated from 15–20 L5-S1 spinal cord sections (separated by 120 μm) in [15]: (1) medial dorsal horn (MDH); (2) lateral dorsal horn (LDH); (3) dorsal commissure (DCM) and (4) lateral laminae V–VII including the sacral parasympathetic nucleus (SPN) in L6-S1 [15]. Counts of Fos-IR cells are presented as average numbers of cells/section or percentage change in numbers of cells/section.

### CGRP immunohistochemistry

Lumbosacral spinal cord sections adjacent to those used for Fos staining (above) were used for CGRP immunohistochemistry. Groups of experimental animals were processed simultaneously to decrease the incidence of variation in staining and background that can occur between animals and on different processing days. Spinal cord sections were processed free-floating as previously described [14]. Briefly, all sections were incubated overnight at room temperature with primary antibody (CGRP; 1:1000; Phoenix Pharmaceuticals, Burlingame, CA) in 1% goat serum and 0.1 M phosphate buffered saline (PBS), and then washed (3 × 10 min) with 0.1 M PBS, pH 7.4. The tissues were then incubated with Cy3-conjugated goat anti-rabbit (Jackson Immunoresearch, West Grove, PA; 1:500) antibody for 2 hours at room temperature. Following washing (3 × 10 min with PBS), spinal cord sections were mounted on gelled (0.5%) slides and coverslipped with Citifluor (Citifluor Ltd., London). Control sections incubated in the absence of primary or secondary antibody were also processed and evaluated for specificity or background staining levels. In the absence of primary antibody, no positive immunostaining was observed.

### CGRP-IR semi-quantification

Density of CGRP-IR in specific regions of the spinal cord was determined with the aid of Meta Morph image analysis software (version 4.5r4; Universal Imaging, Downingtown, PA) as previously described [17,18]. Fluorescence images were converted to a gray scale for the purposes of densitometry. The following regions were analyzed in the L5-S1 spinal cord from both NGF and saline animals: LDH(laminae I and II), MDH, DCM, the region of the SPN (L6-S1), LCP (L6-S1). Of the spinal cord sections processed for CGRP-IR, random sections were selected for densitometry and viewed through a video camera attachment to the microscope. The image was converted to pixels according to a gray scale that ranges in intensity from 0 (white) and 255 (black). The spinal cord section was centered in the field and the midline dorsal septum was located. Then a background level was determined from an area of the spinal cord that did not exhibit CGRP-IR. A background value was used to set the threshold between labeled pixels and background pixels. Illumination intensity was held constant throughout the analysis of NGF and control animals. A standard sized rectangle was overlaid on the areas of interest (DH, DCM, LCP, SPN) and the labeled area within the rectangle was measured. Within each region, four separate measurements were made and averaged. Transmittance (t) was calculated as t = (gray level + 1/256). Optical density (OD) was derived from OD = -log t. Ten-fifteen sections of each segmental level (L5-S1) were examined from each animal. NGF and saline animals that were processed together for CGRP-IR were analyzed together.

### Figure preparation

Digital images with exposure times, brightness and contrast held constant, were obtained using a CCD camera (MagnaFire SP; Optronics; Optical Analysis Corp., Nashua, NH) and LG-3 frame grabber attached to an Olympus microscope. Images were imported into Adobe Photoshop 7.0 (Adobe Systems Incorporated, San Jose, CA).

### Statistics

Comparisons between the number of Fos-IR spinal neurons, NGF bladder content, optical density measurements of CGRP-IR and cystometrogram variables were made using ANOVA. When F ratios exceeded the critical value (p ≤ 0.05), Dunnett's test was used to compare means. All values are expressed as mean ± S.E.M.

## Results

### Continuous NGF infusion into the bladder wall for two-weeks

**Figure 2:**
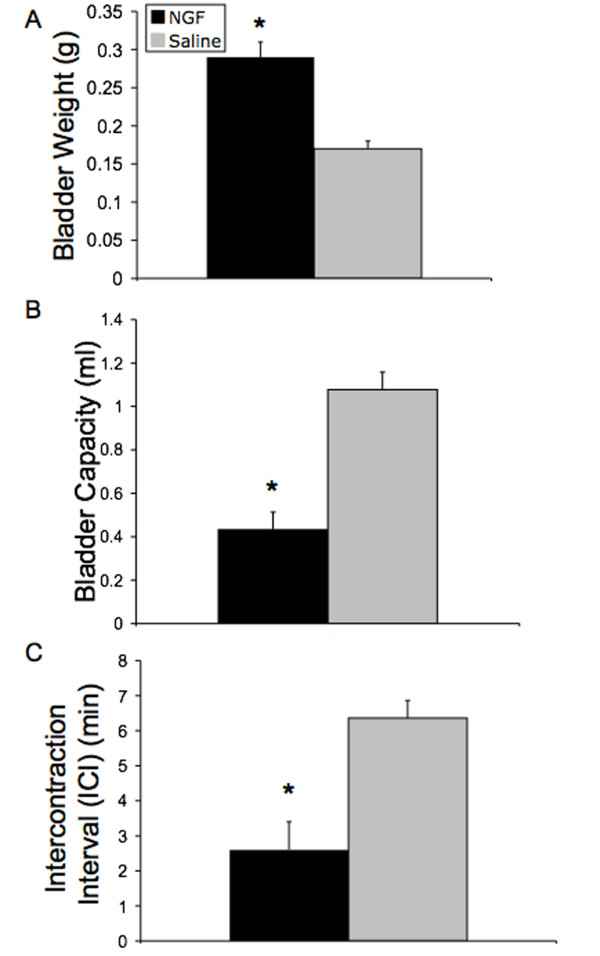
**Effects of NGF on cystometry variables**. Summary bar graphs depict the significant (*, p ≤ 0.01) increase in bladder weight (**A**), decrease in bladder capacity (**B**), and decrease in intercontraction interval (**C**) in NGF-treated rats. 'n' = 6 for each group.

NGF infusion into the bladder wall by osmotic pump significantly increased (p ≤ 0.001; 17-fold) NGF bladder content (Fig. 1D) and bladder mass (Fig. 2A).

### Cystometrograms

**Figure 3:**
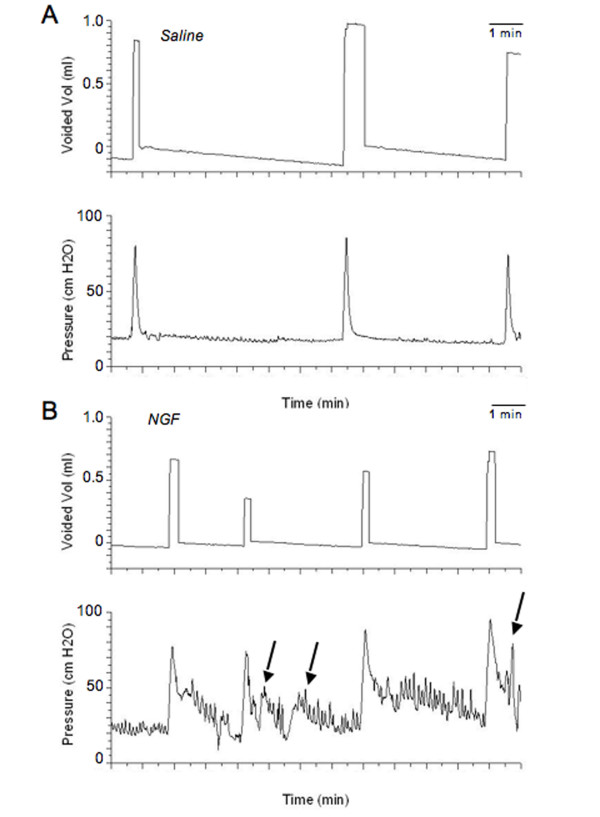
**Cystometrogram recordings**. Exogenous delivery of NGF (2.5 μg/μl) decreased bladder capacity (increased voiding frequency). Continuous cystometrogram recordings in saline (**A**) and NGF-treated rats (**B**). Arrows point to some non-voiding bladder contractions. The x-axis represents the time (minutes, min) and the y-axis represents the intravesical pressure (cm H_2_O). The amount of saline voided (ml) is also illustrated.

**Figure 4:**
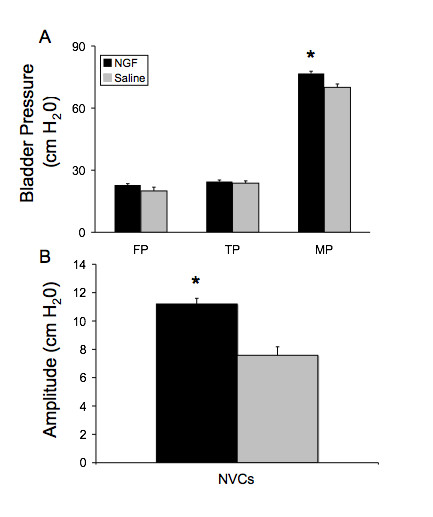
**Effects of NGF on cystometry variables**. Summary bar graphs depict the significant (*, p ≤ 0.05) increase in peak micturition pressure (**B**) and significant (*, p ≤ 0.01) increase in amplitude of non-voiding contractions (NVCs)(**C**) in NGF-treated rats. No changes in filling pressure (FP) or threshold pressure (TP) were observed (**B**). 'n' = 6 for each group.

During bladder filling and micturition, all animals were moving freely in the metabolic cage. No difference in behavior was noted between individual animals or between the study groups. All animals exhibited normal patterns of activity and inquisitiveness. No animals exhibited any outward signs of pain or distress. Infusion of NGF into the bladder wall significantly (p ≤ 0.01) decreased the intercontraction interval (Fig. 2C, Fig. 3) and reduced bladder capacity by 60% (Fig. 2B, Fig. 3). Infusion of NGF into the bladder wall was associated with a significant (p ≤ 0.05) increase in peak micturition pressure but no changes were observed in filling or threshold pressure (Fig. 4A). Continuous bladder filling was associated with non-voiding contractions (NVCs) in four animals in each group (Fig. 3). However, the amplitude of the NVCs was significantly (p ≤ 0.01) greater in the NGF infusion group (Fig. 4B). Inter-animal variability in the frequency of NVCs was clearly present in animals from both groups. PVR was negligible in both groups. No change in bladder compliance was determined for either group.

### Fos protein expression induced by saline distention of the urinary bladder in saline treated rats

**Figure 5:**
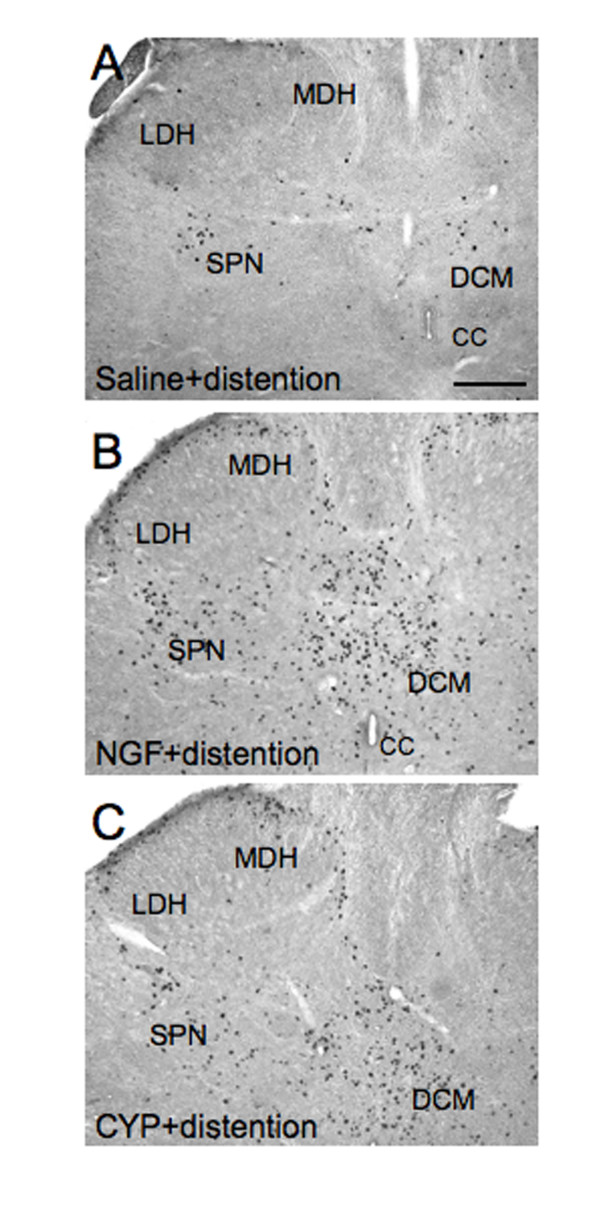
**Fos induction in NGF-treated rats**. Brightfield photographs from sections (40 μm) of the L6 spinal cord showing the distribution of Fos-IR cells after intravesical saline distention (2 hr) in: saline treated rats (**A**), NGF-treated rats (**B**), or cyclophosphamide (CYP) treated rats (**C**). MDH, medial dorsal horn; LDH, lateral dorsal horn; DCM, dorsal commissure; CC, central canal; SPN, sacral parasympathetic nucleus. Calibration bar represents 100 μm.

**Figure 6:**
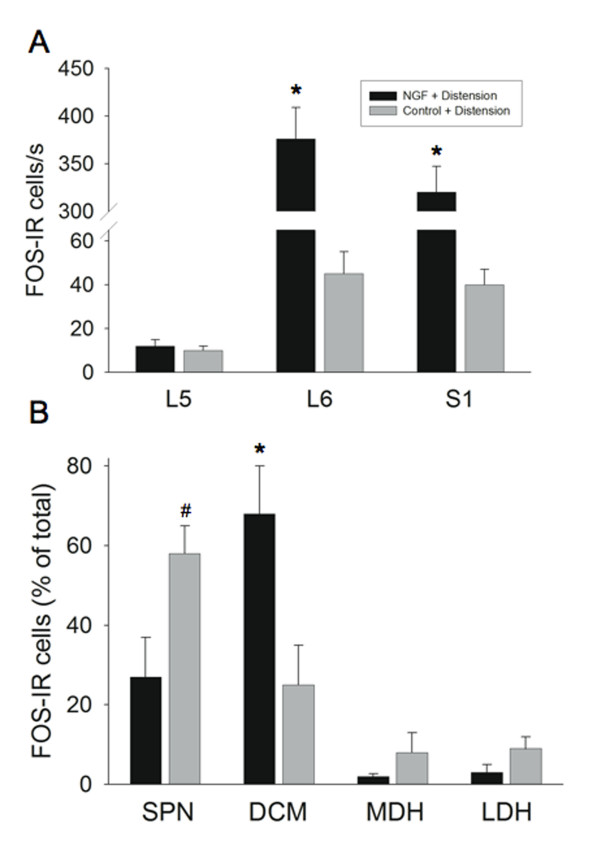
**Magnitude and Distribution of Fos in NGF-treated rats**. Histogram (**A**) showing the segmental distribution of Fos-immunoreactive (IR) cells/section (s) in the rat spinal cord (L5-S1) after intravesical saline distention in saline or NGF-treated rats. *, p ≤ 0.005. Histogram (**B**) showing the distribution of Fos-IR cells in four regions of the L6 spinal cord after intravesical saline distention in saline or NGF-treated rats. Values represent the percentage of the total population of Fos-IR cells induced in each experimental paradigm. The four regions analyzed include: SPN, sacral parasympathetic nucleus; DCM, dorsal commissure; MDH, medial dorsal horn; LDH, lateral dorsal horn. #, p ≤ 0.01; *, p ≤ 0.005. 'n' = 6 for the saline and NGF groups.

In agreement with previous studies [15,19], intravesical saline infusion induced the expression of Fos-IR cells in the L6 (45.0 ± 10.0 Fos-IR cells/section) (Fig. 5, 6A) and S1 (40.8 ± 7.2 Fos-IR cells/section) (Fig. 6A) spinal cord whereas few Fos-IR cells were observed in the L5 spinal segments (Fig. 6A). Fos-IR cells were observed in specific regions of the L6-S1 spinal cord (Fig. 5, 6B). The largest percentage (58 ± 7.5% in L6, 60 ± 6.2% in S1) of Fos-IR cells were observed in the region of the SPN (Figs. 6B). Smaller percentages of Fos-IR cells were present in DCM (25 ± 10.2% in L6, 22 ± 6.6% in S1), MDH (8 ± 5.2% in L6, 9 ± 2% in S1) and LDH (9 ± 3.7% in L6, 9 ± 1.7% in S1) (Fig. 6B).

### Fos protein expression induced by saline distention of the urinary bladder in NGF-treated rats

In rats treated with infusion of NGF into the bladder wall, continuous intravesical infusion (2 hr) of saline significantly (p ≤ 0.005) increased the number of Fos-IR cells observed in the caudal lumbosacral (L6, 376 ± 33.5 cells/section; S1, 320 ± 27.2 cells/section) but Fos protein expression in L5 (12 ± 3.5 cells/section) was not altered (Fig. 5B, 6A). The topographical distribution of Fos-IR cells was also altered (Fig. 5B, 6B). In L6, the majority of Fos-IR cells were distributed in the DCM (68 ± 12.4%) with smaller percentages in the SPN (27 ± 10.0%), MDH (2 ± 0.7%) and LDH (3 ± 2.0%) (Fig. 6B). Similarly in S1, the majority of Fos-IR cells were distributed in the DCM (52 ± 8.8%) with smaller percentages in the SPN (24 ± 6.8%), MDH (15 ± 5.5%) and LDH (9 ± 5.8%).

### Saline distention induced Fos expression in NGF-treated rats compared to saline distention induced Fos expression in CYP-treated rats

The magnitude of Fos-IR cells and the change in the topographical distribution of Fos-IR cells after intravesical saline infusion in NGF-treated animals was similar to that previously reported [15] for CYP-treated rats with continuous intravesical saline infusion (2 hr). In CYP-treated rats with intravesical saline distention (2 hr), the majority of Fos-IR cells in the L6 spinal cord were distributed in the DCM (55.6 ± 7.7%) with smaller percentages in the SPN (27.4 ± 8.5%), MDH (14.2 ± 4.6%) and LDH (2.8 ± 1.6%) (Fig. 5C). The majority of Fos-IR cells in the L6-S1 spinal cord was distributed in the DCM after intravesical saline distention in NGF- or CYP-treated rats.

### Increased expression of CGRP-IR in lumbosacral spinal cord (L6-S1) in NGF-treated rats compared to saline treated rats

**Figure 7:**
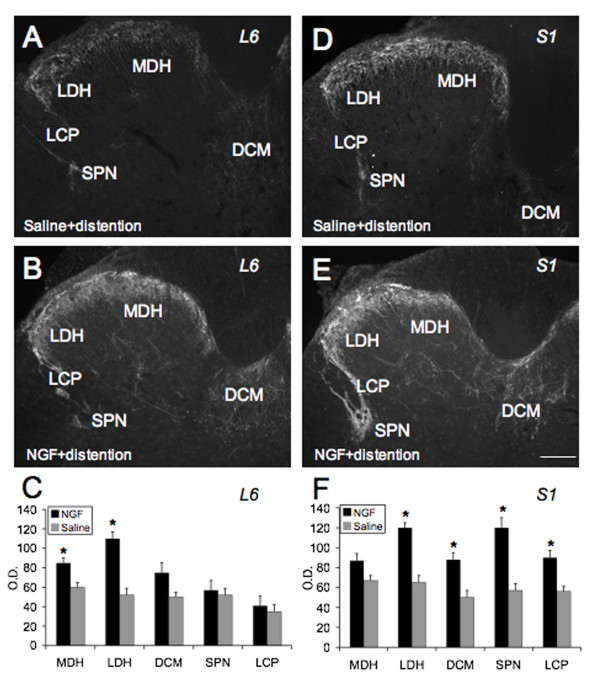
**CGRP Spinal Cord Expression in NGF-treated rats**. CGRP-IR increases in lumbosacral spinal cord with exogenous NGF treatment. CGRP-IR in the L6 (**A**-**B**) and S1 (**D**-**E**) spinal segment in control (**A**, **D**) and NGF-treated (**B**, **E**) rats. **A**, **D**. Fluorescence photographs showing CGRP-IR in the L6 (**A**) and S1 (**D**) spinal segment of control (saline) + bladder distention. **B**, **E**. Fluorescence photographs showing CGRP-IR in the L6 (**B**) and S1 (**E**) spinal segment with NGF treatment + bladder distention. Increased density of CGRP-IR was observed in the medial (MDH) to lateral (LDH) extent of the superficial laminae (I-II) of the dorsal horn (DH) with NGF treatment in L6 (**C**) and S1 (**F**) segments. Changes in CGRP-IR in other spinal cord regions were more dramatic in the S1 spinal segment. Increased CGRP-IR was present in a fiber bundle extending from Lissauer's tract in lamina I along the lateral edge of the DH to the region of the sacral parasympathetic nucleus (SPN) (lateral collateral pathway of Lissauer, LCP) in the S1 segment (**F**). Although this fiber bundle was present in control tissue sections, the staining was less intense (**D**) and was less frequently observed in transverse sections compared to NGF treatment (**E**). Faint CGRP-IR was present in the region of the SPN in control sections (**A**, **D**). With NGF treatment, CGRP-IR in the SPN region also increased in the S1 segment. Increased CGRP-IR was also present in the dorsal commissure (DCM) with NGF treatment (**D**, **E**, **F**). Summary bar graphs of CGRP-IR optical density (O.D.) as measured in specific regions of the L6-S1 spinal cord (**C**, **F**). Calibration bar represents 125 μm. *, p ≤ 0.01.

A number of neuropeptides are present in the somata and processes of urogenital dorsal root ganglion cells with CGRP and substance P being the most widely distributed [20]. In NGF-treated rats undergoing conscious cystometry, significant increases in CGRP-IR in L6-S1 spinal cord regions known to participate in micturition reflexes were observed (Fig. 7). In the L6 spinal segment, CGRP-IR significantly (p ≤ 0.01) increased in the MDH and LDH (Fig. 7A–C). In the S1 spinal segment, CGRP-IR significantly (p ≤ 0.01) increased in the LDH, in the DCM, in the region of the SPN and LCP, through which bladder afferent fibers project to the SPN (Fig. 7D–F). No changes in CGRP-IR were observed in the L5 spinal segment with NGF treatment.

## Discussion

Two-week infusion of NGF into the bladder wall resulted in several novel observations: (1) bladder capacity and intercontraction interval are reduced (~60%) whereas the amplitude of NVCs and bladder weight are increased; (2) Fos protein expression in the L6-S1 spinal cord is increased and the distribution in the lumbosacral spinal cord is altered in response to intravesical saline distention; and (3) CGRP-IR is significantly increased in specific regions of the L6-S1 spinal cord involved in micturition reflexes. These data suggest that NGF infusion into the bladder wall induces bladder overactivity and can reveal a "nociceptive" Fos expression pattern in the spinal cord in response to a non-noxious bladder stimulus. Although other studies have examined the effects of exogenous NGF on bladder function, this study differs from those in several areas: (1) the route of exogenous NGF delivery (intramuscular compared to intrathecal, intravesical or adenovirus-mediated) [5-7]; (2) the demonstration of increased Fos protein and altered Fos distribution induced by bladder distention in NGF-treated rats; and (3) the demonstration of increased CGRP-IR in specific regions of the L6-S1 spinal cord in NGF-treated rats. The present study demonstrates both induction of bladder overactivity and altered spinal cord micturition circuitry in response to exogenous NGF administration.

Neurotrophic factor expression in urinary bladder may underlie neurochemical [13,14], organizational [4,15] and electrical [8] property changes of micturition reflexes after CYP-induced cystitis. The influence of target organ-neuron interactions in the adult animal has been demonstrated [2,4,8]. CYP-induced cystitis alters NGF and receptor expression in urinary bladder, dorsal root ganglia and major pelvic ganglia [16]. NGF scavenging [6,9] reduces bladder overactivity in rats with bladder inflammation.

PBS/IC is a chronic inflammatory bladder disease syndrome characterized by urinary frequency, urgency, suprapubic and pelvic pain [21,22]. Numerous theories including; infection, autoimmune disorder, toxic urinary agents, deficiency in bladder wall lining and neurogenic causes have been proposed Pain and altered bladder function in PBS/IC may involve a change in visceral sensation/bladder sensory physiology. Altered visceral sensations [21,22] may be mediated by changes in peripheral bladder afferent and central pathways such that bladder afferent neurons respond in an exaggerated manner to normally innocuous stimuli (allodynia). Neurotrophins (e.g., nerve growth factor) have been implicated in the peripheral sensitization of nociceptors [23]. Elevated levels of neurotrophins are in urine [10] or in urinary bladder [11] of women with PBS/IC. However, a recent study did not demonstrate an association of increased urothelium/suburothelium NGF with detrusor overactivity [12].

We exogenously delivered NGF into the urinary bladder of control rats and determined effects on bladder function and spinal cord organization of the micturition reflex. Possible sources of NGF and tissues that express TrkA receptor in the inflamed bladder include the urothelium, inflammatory cell infiltrates, and detrusor smooth muscle [4,16]. Acute (30 min) intravesical infusion (20 μg/ml) [6], or chronic intrathecal (1–2 weeks, 2.5 μg/μl) [5] infusion of NGF induce bladder overactivity. We chose to infuse NGF into the bladder wall for a two-week period (2.5 μg/μl) to determine if this route and duration of infusion, used for intrathecal administration [5], could also induce bladder overactivity. This dose and duration of NGF delivery produced a NGF bladder content of 15 pg/μg protein. This NGF level was comparable to those reported in tissues (bladder, spinal cord) after spinal cord injury [16] or bladder outlet obstruction [2]. With NGF treatment in the present study, rats exhibited bladder overactivity with a reduction in bladder capacity and increased frequency of voiding (decreased intercontraction interval). Suturing PE tubing directly into the bladder wall for a focused delivery likely contributed to the appearance of NVCs in 4/6 animals in both control and NGF groups. We do not normally observe NVCs in control rats with implanted intravesical catheters [3,9]. Despite the appearance of NVCs in both groups, the amplitude of the NVCs in the NGF-treated group was significantly greater. We also observed a small, but significant, increase in peak micturition pressure with NGF treatment but the reasons for this are not clear. An increase in peak micturition pressure may result from changes in urethral outlet resistance resulting in increases in PVR. However, PVR was negligible in both NGF-treated and control rats. It should also be noted that NGF levels achieved in the present study were less than those achieved in the DRG [5] with intrathecal infusion of NGF at the identical dose and duration used in the present study. Despite lower NGF tissue levels in bladder compared to DRG [5], bladder overactivity was still observed.

The present study also sought to examine the effect of exogenous NGF treatment on Fos protein expression induced by bladder distention to determine if bladder overactivity was associated with altered (magnitude and distribution) Fos expression in L6-S1 spinal neurons. NGF infusion in the bladder wall increased expression of Fos-IR in spinal neurons and altered the Fos expression pattern in the caudal lumbosacral spinal cord induced by bladder distention. Intravesical saline distention induces Fos-IR in spinal neurons located predominantly in the MDH, LDH and SPN regions of L6-S1 spinal cord of the control animals [15,19]. The pattern and magnitude of Fos expression induced by intravesical saline distention is altered with NGF treatment. Saline or NGF treatment did not affect threshold or filling pressure and there was a small, but significant effect on peak micturition pressure. Thus, it is unlikely that increases in urinary bladder pressure contribute substantially to increased expression of Fos protein. The altered Fos distribution pattern in NGF-treated rats with intravesical saline distention resembles that following noxious irritation (1% acetic acid) [19] of urinary bladder in control animals and intravesical saline (non-noxious) bladder distention in CYP-treated rats [15]. Thus, NGF treatment with intravesical saline distention can reveal a "nociceptive" Fos expression pattern in the spinal cord in response to a non-noxious bladder stimulus. This situation may be analogous to the altered visceral sensation of PBS/IC patients in which a normally non-noxious stimulus (bladder filling) is perceived as painful [21,22]. Altered visceral sensations from the urinary bladder (i.e., pain at low or moderate bladder filling) that accompany PBS/IC [21,22] may be mediated by changes in the properties of peripheral bladder afferent and central pathways such that neurons respond in an exaggerated manner to normally innocuous stimuli (allodynia).

The present study determined if exogenous NGF treatment could affect the central distribution of the neuropeptide, CGRP, known to be expressed in bladder afferent cells in the dorsal root ganglia as well as in afferent nerve processes. Several studies have demonstrated alterations in neuropeptide and neuropeptide receptor expression in PBS/IC and in animal models of bladder inflammation. Substance P (SP)-IR [24] and SP receptor (neurokinin-1) mRNA [25] increases in bladder biopsies from patients with IC. Rodent models of bladder inflammation using either intravesical mustard oil [26] or intraperitoneal injection of cyclophosphamide [14] have demonstrated increases in CGRP- and SP-IR in bladder afferent neurons and central projections. The functional significance of an up-regulation of CGRP in bladder pathways following CYP-induced cystitis is not known but changes in neuropeptide expression and release at both central and/or peripheral afferent terminals is possible. Several reports have suggested that neuropeptide-containing, capsaicin-sensitive bladder afferents may mediate urinary bladder overactivity [27-29]. Botulinum toxin continues to be explored as a novel treatment for IC/PBS and sensory urgency [30,31]. Several studies suggest that improvements in bladder function and reductions in bladder pain responses are attributable to botulinum toxin inhibition of CGRP release from afferent nerve terminals [30,31]. In addition to potential effects of CGRP on bladder function, changes in neuropeptide expression and release at central terminals could further result in a remodeling of spinal cord circuitry controlling micturition [32]. This remodeling may include changes: (1) in the synaptic organization of spinal micturition reflexes; (2) in the neurochemical coding of specific neuronal elements (primary afferent neurons, interneurons) and (3) in the organization of ascending and descending projections to spinal reflexes. In addition to neurochemical changes in micturition reflex circuitry, changes in the electrical [5,8] properties of neurons involved in micturition reflexes after bladder inflammation have also been demonstrated. Such changes may also be mediated by altered expression of neurotrophic factors [5,8] and channel proteins [8] and may underlie bladder functional changes observed with bladder overactivity.

## Conclusion

Exogenous infusion of NGF into the bladder wall reduces the intercontraction micturition interval, reduces bladder capacity, increases bladder mass, increases and changes the Fos protein expression in the L6-S1 spinal cord and increases CGRP expression in specific regions of the L6-S1 spinal cord. The present study adds further support for the involvement of urinary bladder NGF in bladder overactivity and altered central micturition circuitry.

## List of abbreviations

NGF- Nerve growth factor.

PBS- Painful bladder syndrome.

IC- Interstitial cystitis.

LUT- Lower urinary tract.

CYP- Cyclophosphamide.

MDH- Medial dorsal horn.

LDH- Lateral dorsal horn.

DCM- Dorsal commissure.

SPN- Sacral parasympathetic nucleus.

NVCs- Non-voiding contractions.

LCP- Lateral collateral pathway.

PVR- Post void residual.

CGRP- Calcitonin gene-related peptide.

SP- Substance P.

## Competing interests

The author(s) declare that they have no competing interests.

## Authors' contributions

MAV designed these studies, analyzed, interpreted data and supervised the work by laboratory technicians and associates. MAV assisted in the performance of the Fos, CGRP studies and NGF ELISAs. MAV wrote the manuscript and has given final approval for the manuscript to be published. MAV obtained the NIH funding for these studies.

PZ assisted in the design of the NGF studies, performed the NGF studies, analyzed and interpreted the NGF data. PZ reviewed and revised drafts of the manuscripts. PZ has given final approval for the manuscript to be published.
